# Recent Advances in Software Tools for More Generic and Precise Intact Glycopeptide Analysis

**DOI:** 10.1074/mcp.R120.002090

**Published:** 2021-02-06

**Authors:** Weiqian Cao, Mingqi Liu, Siyuan Kong, Mengxi Wu, Yang Zhang, Pengyuan Yang

**Affiliations:** 1 The Fifth People’s Hospital of Fudan University and Institutes of Biomedical Sciences, Fudan University, Shanghai, China; 2 NHC Key Laboratory of Glycoconjugates Research, Fudan University, Shanghai, China; 3 The Shanghai Key Laboratory of Medical Epigenetics and the International Co-laboratory of Medical Epigenetics and Metabolism, Ministry of Science and Technology, Fudan University, Shanghai, China; 4 Department of Chemistry, Fudan University, Shanghai, China

**Keywords:** software tools, intact glycopeptide analysis, mass spectrometry, bottom-up experimental strategies, quality control, software applications, CID, collision-induced dissociation, ETD, electron-transfer dissociation, FDR, false discovery rate, HCD, higher-energy collision dissociation, PD, product dependent, Q-TOF, quadrupole-time of flight, SCE, stepped collision energy

## Abstract

Intact glycopeptide identification has long been known as a key and challenging barrier to the comprehensive and accurate understanding the role of glycosylation in an organism. Intact glycopeptide analysis is a blossoming field that has received increasing attention in recent years. MS-based strategies and relative software tools are major drivers that have greatly facilitated the analysis of intact glycopeptides, particularly intact N-glycopeptides. This article provides a systematic review of the intact glycopeptide-identification process using MS data generated in shotgun proteomic experiments, which typically focus on N-glycopeptide analysis. Particular attention is paid to the software tools that have been recently developed in the last decade for the interpretation and quality control of glycopeptide spectra acquired using different MS strategies. The review also provides information about the characteristics and applications of these software tools, discusses their advantages and disadvantages, and concludes with a discussion of outstanding tools.

## Highlights

• Summarize the development of software tools for intact glycopeptide analysis.
• Survey the MS acquisition strategies for glycopeptide spectrum acquisition.
• Conclude the quality control methods for glycopeptide-spectrum matching.
• Show the application of intact glycopeptide analysis in glycoproteomic research.

## Graphical abstract

**Figure:**
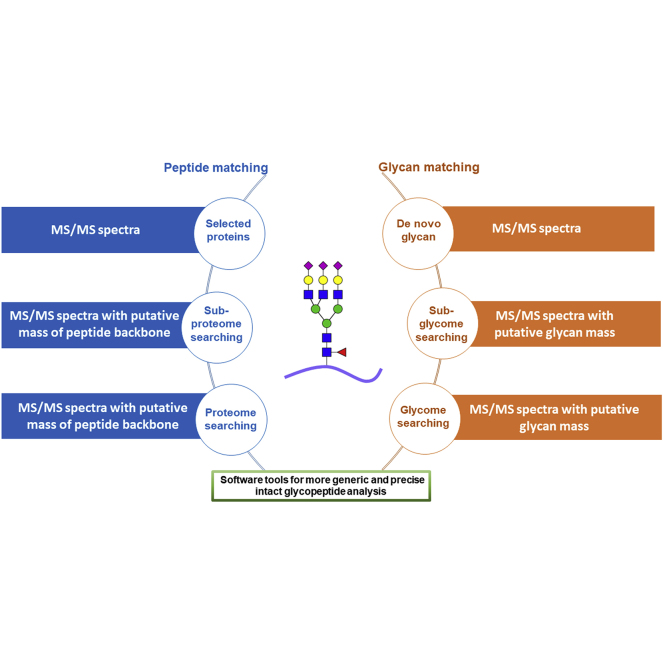

## In Brief

This article provides a systematic review of the most recent MS-based strategies and corresponding software tools for the analysis of intact glycopeptides, particularly intact N-glycopeptides, reported in the last decade, including the process of identifying N-glycopeptides from MS data, the existing methods of MS data acquisition and interpretation, the quality control methods, the display of results, and the software applications.

Protein glycosylation has long been known as a heterogeneous post-translational modification generating greater protein diversities than other post-translational modifications (1). These diversities alter the functions of proteins and subsequently exert a profound effect on various biological processes (2, 3). The ability to perform an in-depth and precise identification of glycoproteins is the key to obtaining a comprehensive and accurate understanding of the role of glycosylation in an organism.

MS has been recognized as an ideal tool for glycoprotein analysis since the first report in 1978 (4). MS-based glycoproteomics, which emerged with the goal of defining the glycoproteome of a biological system, has made strides in deglycosylation-centric glycoproteomic studies in the past few decades (5, 6, 7, 8, 9). Previously, enriched glycopeptides were usually deglycosylated to generate an identifiable mass tag on glycosylation sites before the MS analysis that can be directly searched using routine proteomic software. Although the use of the deglycosylation-centric strategy as a compromise greatly simplified the MS identification and expanded our knowledge of glycoproteins, the site-specific glycan information was lost during the removal of glycans from glycopeptides. Subsequently, a more challenging issue in the high-throughput and precise identification of intact glycopeptides (also known as site-specific glycan analysis) was identified to better assess the biological attributes of the glycosylation.

Since then, an analysis of intact glycopeptides has been a challenge for several reasons. First, the inherently low abundance and ionization efficiency of glycopeptides make them difficult to identify using MS. Second, MS dissociation energy for glycans and peptides differ, hampering the acquisition of informative glycopeptide spectra using the single MS fragmentation method. Third, glycopeptides, which comprise a group of various types of monosaccharides linked to amino acid residues with different compositions and linkages, are so complicated that routine proteomic search engines are not qualified for an analysis of such enormous numbers of variable modifications. Many robust and mature glycopeptide enrichment methods developed in the past decades have dramatically improved the abundance of glycopeptides in MS analysis and considerably reduced the signal suppression of glycopeptide in the mass spectra by nonglycopeptides. Subsequently, the urgent needs for intact glycopeptide analysis are the ability to obtain informative glycopeptide spectra data and reliably decipher those data with expert software.

In recent years, substantial progress has been achieved in addressing the immediate needs for an analysis of intact glycopeptides, particularly N-glycopeptides (10). Some generic analytical pipelines and useful software became available and enabled a high-throughput and precise analysis of intact glycopeptides. Through intact glycopeptide analyses, glycopeptide sequences with attached glycan composition can be conceivably gained. This review will specify the most recent MS-based strategies and corresponding software tools for the analysis of intact glycopeptides, particularly intact N-glycopeptides, reported in the last decade, while reviewing the process of identifying N-glycopeptides from MS data, the existing methods of MS data acquisition and interpretation, the quality control methods, the display of results, and the software applications (Table 1).

**Table 1:** A summary table of the most recent MS-based strategies and corresponding software tools for the analysis of intact N-glycopeptides

Pipeline	Spectral collection				Scoring/matching					Quality control		
	MS/MS			MS^3^/de- glycopeptide	Peptide		Glycan		Diagnostic ions	Peptide	Glycan	Intact glycopeptide
	HCD	CID	ETD		Method	Database	Method	Database				
GlycoFragwork, etc. (Tang's laboratory)	BY-C	Y-a	c/z		Probability matches	Selected proteins	*De novo*		Glycan classification	Decoy sequence		
GlycoPep Detector, etc. (Desaire's laboratory)		Y-a	c/z		Probability matches	Selected proteins	Probability matches	Manual input		Decoy sequence		
GlycoPeptide Search		Y-a b/y			Existing algorithm	Selected proteins	Core Y-ions matches	GlycomeDB	Spectral filtration	Decoy sequence		
GP Finder	Y-a				Probability matches	Selected proteins	Probability matches	Glycomic analysis	Spectral filtration			Decoy precursor mass
Sweet-Heart, etc. (Khoo's laboratory)	BY-c	Y-a	c/z	b/y from MS^3^	Existing algorithm	Proteome	Probability matches	Semi de-novo	Product-trigger	Decoy sequence		
ArMone	Y-c			De-glyco needed^b^	Mass compare	Sub- Proteome^e^	Core Y-ions matches	GlycomeDB				
GRIP	Y-c	Y-a		De-glyco needed^b^	Mass compare	Sub- Proteome^e^	Probability matches	GlycomeDB				
GPQuest	Y-a			De-glyco needed^c^	Spectral library	Sub- Proteome^e^	Mass compare	Glycomic analysis	Spectral filtration	Decoy sequence		
GlycoFinder	BY-a				Mass compare	Selected proteins	Core Y-ions matches	Manual input	Spectral filtration			
GlycoMaster DB	Y-a		c/z		Existing algorithm	Proteome	Probability matches	GlycomeDB	Spectral filtration	Decoy sequence		
MAGIC	B b/y Y-a				Existing algorithm^d^	Proteome	Core Y-ions matches	Semi de-novo	Spectral filtration	Decoy sequence		
I-GPA, etc. (Yoo's laboratory)	B b/y Y-a				Probability matches	Sub- Proteome^f^	Probability matches	GlycomeDB	Glycan classification			Decoy glycopeptide
GlycoPAT	b/y	Y-a	c/z		Probability matches	Selected proteins	Probability matches	Semi de-novo	Spectral filtration			Decoy glycopeptide
SugarQB	B b/y Y-a				Existing algorithm^d^	Proteome	Core Y-ions matches	Manual input	Spectral filtration	Decoy sequence		
glyXtoolMS	B b/y Y-a				Probability matches	Selected proteins	Core Y-ions matches	GlyTouCan	Glycan classification			
pMatchGlyco	B b/y Y-a			De-glyco needed^c^	Spectral library	Sub- Proteome^e^	Mass compare	GlycomeDB				Decoy glycopeptide
GPSeeker	b/y Y-a				Existing algorithm	Proteome	Probability matches	Glycomic analysis	Spectral filtration	Decoy sequence	Decoy fragment^g^	
O-Search	BY-c				Existing algorithm^d^	Proteome	Core Y-ions matches	Semi de-novo	Spectral filtration	Decoy sequence		
pGlyco	BY-c	Y-a		b/y from MS^3^	Existing algorithm	Proteome	Probability matches	GlycomeDB	Product-trigger	Decoy sequence	Decoy fragment	
pGlyco 2.0	B b/y Y-a	Analyzed^a^			Existing algorithm	Proteome	Probability matches	GlycomeDB	Spectral filtration	Decoy sequence	Decoy fragment	Comprehensive probability

CID, collision-induced dissociation; ETD, electron-transfer dissociation; HCD, higher-energy collision dissociation; Y-a, all Y ions; Y-c, Y ions from N-glycan core.

a Different collisional energies.

b Pregenerated de-glycopeptide database.

c Pregenerated de-glycopeptide spectra.

d Substitution of precursor mass.

e Candidates from de-glyco experiment.

f Candidates from the sample-dependent database.

g The decoy method from pGlyco.

## MS Fragmentation Methods for Glycopeptides

In the past 10 years, software tools for the automatic identification of intact glycopeptides using different LC-MS/MS strategies were developed. Regardless of the type of strategy used, sufficient fragments from both peptide backbones and the attached glycans are required. Informative glycopeptide mass spectra are difficult to acquire because the fragmentation varies widely between the two components because of the differences in the physical and chemical properties of the glycosidic bonds and peptide bonds. Dissociation methods, including resonance activation (ion trap) collision-induced dissociation (CID), beam-type (quadrupole-time of flight [Q-TOF]) CID, higher-energy collision dissociation (HCD), and electron-transfer dissociation (ETD), are the most commonly used approaches in glycopeptide identification.

### CID Fragmentation

Mass spectra generated by ion-trap CID are dominated by Y-ions but contain a few B-ions and a limited number of b/y-ions because of the single-bond cleavages of precursor ions, low dissociation energy, and the “1/3” cutoff in the low m/z region (11), which can be used to identify the conjugated glycans but not for confident identification of the location of the glycosylation site and peptide sequences, particularly in complex samples. Thus, this technique has only been applied to analyze glycopeptides in simple samples in some previous studies (12, 13, 14, 15).

### HCD Fragmentation

In contrast, HCD tends to produce abundant diagnostic oxonium ions, such as m/z 138.055, 204.087 and 366.140 fragments. It also yields B- and Y-ions for glycan identification and partial b/y-ion series for peptide identification. Studies showed an increase in the b/y-ion series as the HCD collision energy increased (16). Different collision energies in HCD-MS/MS produce complementary fragments of the glycan and peptides (17, 18). GlycoFinder used HCD collision energies of 30% and 50% to analyze glycopeptide in simple samples (19). The fragmentation patterns produced by the beam-type CID in the Q-TOF spectra resemble the patterns produced by HCD in C-trap on Orbitrap mass spectrometers.

Many studies have investigated the behaviors of glycopeptide fragmentation using different dissociation methods (20, 21, 22, 23, 24, 25). In 2016, we used various MS/MS collision parameters, including CID and HCD, each with nine different energies, as well as electron-transfer/collision-induced dissociation/electron-transfer/higher-energy collision dissociation. Stepped collision energies (SCEs)–HCD–MS/MS at 20-30-40% energies generated the most informative and abundant fragment ions for both the glycan and peptide of a glycopeptide in a single spectrum (26). Since then, SCE–HCD–MS/MS has been widely used in high-throughput identification of intact glycopeptides (27, 28).

### ETD Fragmentation

ETD primarily breaks N-C_α_ bonds and generates c/z-ions. Ideally, ETD generates abundant c/z-ions from peptide backbone fragments, which are very useful for the identification of glycosylation sites and peptide sequences. However, ETD often suffers from incomplete fragmentation, leading to a large amount of residual precursor ions (29). Thus, ETD is rarely used alone and is frequently combined with HCD or CID methods for glycopeptide identification. With the development of the ion-trigger technique in MS, HCD–product-dependent (PD)–ETD increases the efficiency of combining fragments because ion-trigger technology generally avoids the reaction time of ETD wasted on nonglycopeptide dissociation. Hybrid dissociation techniques developed for an Orbitrap Fusion mass spectrometer, such as electron-transfer/collision-induced dissociation (30, 31), electron-transfer/higher-energy collision dissociation (29, 32, 33), and activated ion electron transfer dissociation (34, 35), where a supplementary energy source is applied to all fragment ions formed by ETD, have shown great potential in N-glycopeptide identification (11, 35, 36, 37, 38, 39).

## Bottom-Up Experimental and MS Acquisition Strategies

In a typical bottom-up intact glycopeptide-identification strategy, glycopeptides are first enriched to reduce the complexity and increase the detectability of glycopeptides. Then, enriched intact glycopeptides are analyzed using different LC-MS/MS strategies and interpreted with different software tools. After surveying the development of software tools for MS-based glycopeptide-identification strategies in last 10 years, two major types of software have been used according to the experimental strategy with or without a deglycosylation procedure: one is the software combining the results from two LC-MS/MS analyses (one with deglycosylated peptides and another with native intact glycopeptide) and the other is the software that directly identifies mass spectra from an intact glycopeptide analysis.

### Experimental and MS Acquisition Strategy With the Deglycosylation Procedure

Generally, in the experimental strategy with the deglycosylation step, two experimental workflows, deglycosylated peptide analysis and intact glycopeptide analysis, are performed. A spectral library of peptides containing glycosylation sites in the samples is first built by analyzing the isolated deglycosylated peptides using HCD/CID LC-MS/MS. Spectra of intact glycopeptides are selected using diagnostic oxonium ions. The glycan composition at a glycosylation site is determined by matching the mass difference between the precursor ion of the intact glycopeptide and the deglycosylated peptide to the glycan database. Three main software tools support this identification strategy: GRIP developed by us in 2014 (40), ArMone 2.0 developed by Cheng *et al.* in 2014 (41), and GPQuest developed by Toghi Eshghi *et al.* in 2015 (42). Although GRIP, ArMone, and GPQuest have been used for high-throughput profiling of intact N-glycopeptides in complex samples, the two workflows make the experimental operation tedious. GlycoSeq developed by Yu *et al.* in 2016 (43), which aims to identify glycopeptides in isolated glycoproteins from their CID spectra alone, also requires a preliminary step for identifying the deglycosylated sites.

### Experimental and MS Acquisition Strategy Without the Deglycosylation Procedure

In the experimental strategy without the deglycosylation step, software tools were developed to directly identify intact glycopeptides in mass spectra. The software tools were designed either for the interpretation of MS/MS data from one type of fragmentation method or for MS/MS data from multiple fragmentation methods. In early years, many software tools were expertly designed for a specific dissociation-based LC-MS/MS analysis of glycopeptides. For example, GlycoPeptide Search (14, 15), GP Finder (12), and MAGIC (44) were all developed for a Q-TOF–CID MS/MS analysis, in which GP Finder was specifically designed for a nonspecific analysis of digested glycopeptides using Pronase. ProteinProspector (45, 46) and GlycoPep Detector (47) were designed for an ETD–MS/MS analysis. GlycoPep Grader (13), which was developed by the same laboratory as GlycoPep Detector, was used for CID–MS/MS analyses. Although the experimental procedures of these software-supported analytical strategies are easy to operate, information about both glycan and peptide fragments in a spectrum obtained from a single dissociation method is insufficient and substantially limits the throughput and reliability of the identification. All of these software tools, except MAGIC (44), are only suitable for an analysis of simple samples.

Combined dissociation methods have been applied to analyze glycopeptides and obtain informative glycopeptide mass spectra. HCD, ETD, and ion-trap CID are often combined to dissociate the same precursor ions because of their complementary nature when applied to N-glycopeptide dissociation. Corresponding software tools were developed for deciphering a precursor with multiple fragment types in MS/MS data. Byonic, which is a commercial and most commonly used software (48), supports searching MS/MS data with multiple fragment types. gFinder (49) and I-GPA (50) developed by Yoo *et al.* were both designed to interpret CID/HCD–MS/MS spectra. GlypID 2.0 improves upon the GlypID tool (51) developed by Tang *et al.* and supports searches for HCD and CID fragmentation ions. GlycoFragwork developed by the same laboratory characterizes glycopeptide in biological proteome samples by scoring complementary fragmentation techniques, including CID, HCD, and ETD. GlycoPAT (52) supports searches of CID/ETD–MS/MS or CID/HCD/ETD–MS/MS data. GlycoMaster DB (53) was designed to decipher HCD/ETD combined with MS/MS data and HCD-MS/MS data alone. Sweet-Heart, a series of software tools developed by Khoo *et al.* substantially improved the analytical procedure of glycopeptide identification from multiple fragmentation mass data. Sweet-Heart was first proposed in 2013 as an integrated suite of computational tools enabling the sequencing and identification of glycopeptides from low-resolution and low accuracy CID-MS^2^ and targeted-CID-MS^3^ (54). In 2014, taking advantage of the new trihybrid Orbitrap configuration, the authors experimented with adding a parallel ion-trap CID-MS^2^ data acquisition mode to the HCD-PD-ETD function and optimized Sweet-Heart for this analytical procedure (55).

The combination of HCD/CID with ETD is one of the widely used methods in multiple dissociation strategies. However, the long reaction time and low dissociation efficiency of ETD have limited the throughput of glycopeptide analysis. Recently, a preferable strategy using SCE-HCD-MS/MS and a dedicated search engine pGlyco 2.0 (26) to completely utilize the abundant information in SCE-HCD-MS/MS spectra were developed, enabling a high-throughput analysis of N-glycopeptides.

## Interpretation of Glycopeptide Mass Spectra

The method used to interpret complex tandem mass spectra generated from glycopeptides is the core of a search tool. Generally, processes used to interpret glycopeptide mass spectra include screening glycopeptide mass spectra and matching the glycan moiety and carrier peptide with glycopeptide spectra.

### Selection of Glycopeptide Mass Spectra

A screen of glycopeptide mass spectra is preferred to avoid interference from nonglycosylated peptide spectra and improve the reliability of the identification. The abundant glycopeptide-specific oxonium ions obtained upon HCD are normally used to determine whether the spectrum is attributed to a glycopeptide. The HexNAc-derived fragment ions m/z 138.055, m/z 204.087, and m/z 366.140 derived from Hex+HexNAc are mostly used as diagnostic ions for the selection of glycopeptide spectra. These diagnostic ions have been utilized either “online” in the “HCD-PD” mode to enable a more efficient time of dissociation of glycopeptides instead of nonglycopeptides in the subsequent triggered ETD or CID or “offline” as a diagnostic ion assistant for the software-based selection of glycopeptide spectra. In addition, fragment ions m/z 292.103/274.092 and m/z 308.098/290.087 for NeuAc/NeuAc-H_2_O and NeuGc/NeuGc-H_2_O, respectively, are specific for sialic acid–containing glycopeptides. Other diagnostic oxonium ions, such as m/z 109.028, m/z 115.035, m/z 127.039, and 145.050 from fragments of Hex, m/z 126.055, m/z 144.066, m/z 168.066, and m/z 186.076 from fragments of HexNAc, m/z 657.140 from Hex+HexNAc+NeuAc, and m/z 673.230 from Hex+HexNAc+NeuGc, are also used alone or in combination for either the selection of glycopeptide spectra or glycan matching.

Another strategy for selecting glycopeptide spectra is based on precursor ions (56, 57, 58). Isotope-targeted glycoproteomics (IsoTag), in which metabolic labeling of glycoproteome is combined with the chemical enrichment and isotopic recoding of glycopeptides with a dibromide motif developed by Woo *et al.*, (58) is the most representative approach for that strategy. The natural abundance of the stable isotopes, ^79^Br and ^81^Br (1:1), were used in the study by Palaniappan *et al.* (57) to provide a ready source for isotope recoding and convoluting the mass envelope of a glycopeptide with a triplet signature (1:2:1(M, M + 2, M + 4)), which can be detected computationally using a pattern-recognition algorithm developed in-house.

### Matching of the Glycan Moiety and Carrier Peptide

**Fig. 1:**
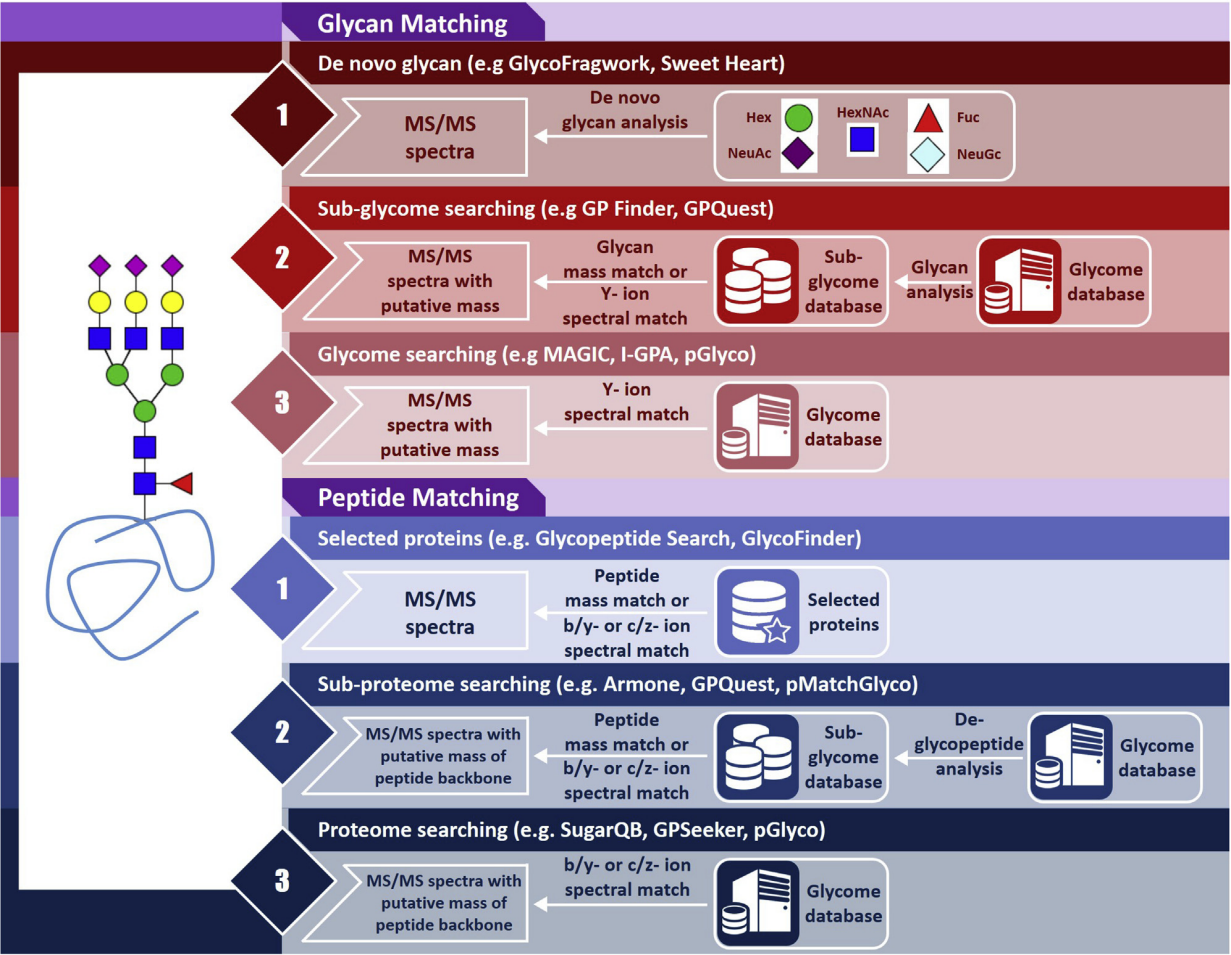
**General approaches for matching of the glycan moiety and carrier peptide**.

Computational tools tackle the matching of the glycan moiety and carrier peptides using different approaches probably according to the different experimental and MS acquisition strategies they used (Fig. 1). Generally, for an analysis of a glycan moiety, most tools perform probability matches against the glycan database from GlycomeDB (currently merged into GlyTouCan). A few tools developed semi *de novo* glycan profiling algorithms; for carrier peptide analysis, software tools perform probability matching against select protein sequences. When a complete proteome database is used in a large-scale analysis, the incorporation of existing proteomic search engines (*i.e.*, Mascot, Sequest, and pFind) is a routine approach (59, 60).

The approach for the strategy with the deglycosylation step considers the carrier peptide as a predefined mass increment according to their pre-established spectral library of peptides containing glycosylation sites to deduce the glycan moiety from glycopeptide spectra with different glycan matching strategies. The core of GRIP is a network-centric algorithm for deciphering glycan fragmentation CID MS/MS spectra (40). ArMone 2.0 identifies glycans by searching a constructed glycan database with a calculated molecular weight of glycans and MS/MS spectra of the glycopeptide, while only scoring the pentasaccharide core structure observed in the N-glycosylation (Man_3_GlcNAc_2_) of mammalian proteins. GPQuest assigns the glycan composition by matching the mass difference between the precursor ion of the intact glycopeptide and the glycosite-containing peptide to a glycan database (42). It is practically impossible to distinguish two glycan compositions with the same molecular weight when deducing the glycan moiety based only on the molecular weight. The same limitation also exists when directly matching the carrier peptide from the glycopeptide spectra with the peptide library, although common retention times have been used to filter between the two experiments (identification of the intact glycopeptide and deglycosylated peptide) in ArMone 2.0.

The interpretation approach for the strategy using combined fragmentation methods without the deglycosylation step generally assigns the glycan composition from acquired CID-MS/MS spectra that are rich in glycan fragment ions and define the carrier peptide sequences by an additional mode of MS^2^ or MS^3^ analysis (61). The corresponding computational tools mentioned in the “Experimental and MS Acquisition Strategy Without the Deglycosylation Procedure” section all use this approach to deduce the glycan composition and peptide sequences of glycopeptides. For example, Sweet-Heart identifies the glycan composition in low-resolution and low-accuracy ion-trap MS^2^ data and putative peptide backbone in targeted MS^3^ data (54).

The approaches used by software tools to interpret the single fragmentation patterns differ. Some mandatorily detect the glycan and peptide ions in one spectrum. Meanwhile, other search tools, such as MAGIC (44) and SugarQB (62) for HCD/Q-TOF spectra interpretation, detect Y-ion patterns from the fragmentation of the common trimannosyl core of N-glycopeptides, and search the subsequent peptide sequence using common database search engines, such as Mascot, through the generation of *in silico* spectra by overwriting the original precursor with the naked peptide m/z and removing all of the glycan-related ions.

A superior interpretation strategy was used by pGlyco 2.0 (26), which was developed for the interpretation of abundant information in SCE-HCD-MS/MS spectra. It performed an integrated open search of each spectrum: a spectrum was first scored against the glycome database to identify the glycan candidates and then scored against the proteome database to identify the peptide candidates. This process substantially increased the efficiency and accuracy of the interpretation.

## Quality Control Methods for Glycopeptide-Spectrum Matching

The false discovery rate (FDR) is an important measurement and a crucial parameter used to define the specificity of the identification and quality control of results. Since this technique was first proposed by Gygi in proteomics (63, 64), the target-decoy approach has been adopted as an acceptable method to calculate the FDRs for glycopeptides.

Before 2016, almost all identification software estimated the FDR of glycopeptide identification only at the peptide level by using a decoy database created based on the target protein sequence database. For example, GPQuest (42) creates a decoy database by randomly arranging amino acid sequences of those glycosite-containing peptides identified in their two-step experimental strategy with the same length as the target database. GlycoPep Evaluator (65) is a software program designed for the FDR analysis of small datasets that generates a decoy database with fixed mass of attached glycan for each glycopeptide in a 1:20 target-to-decoy ratio, according to a user-specified mass tolerance interval.

However, a high FDR would be obtained for glycopeptide identification results even if the peptide-spectrum match score is high because the FDR is only calculated at the peptide level and does not control for glycan identification. For example, Wu *et al.* (55) manually compared the results of HCD spectra identified using Sweet-Heart and Byonic (48) and validated as high as 37% false positives from 551 positive spectrum matches despite a claim of zero FDR using the Byonic criteria because they lack an FDR estimation of the glycan identity. As shown in the study by us, Byonic might substantially underestimate the metric of FDR for glycopetide identification (26).

**Fig. 2:**
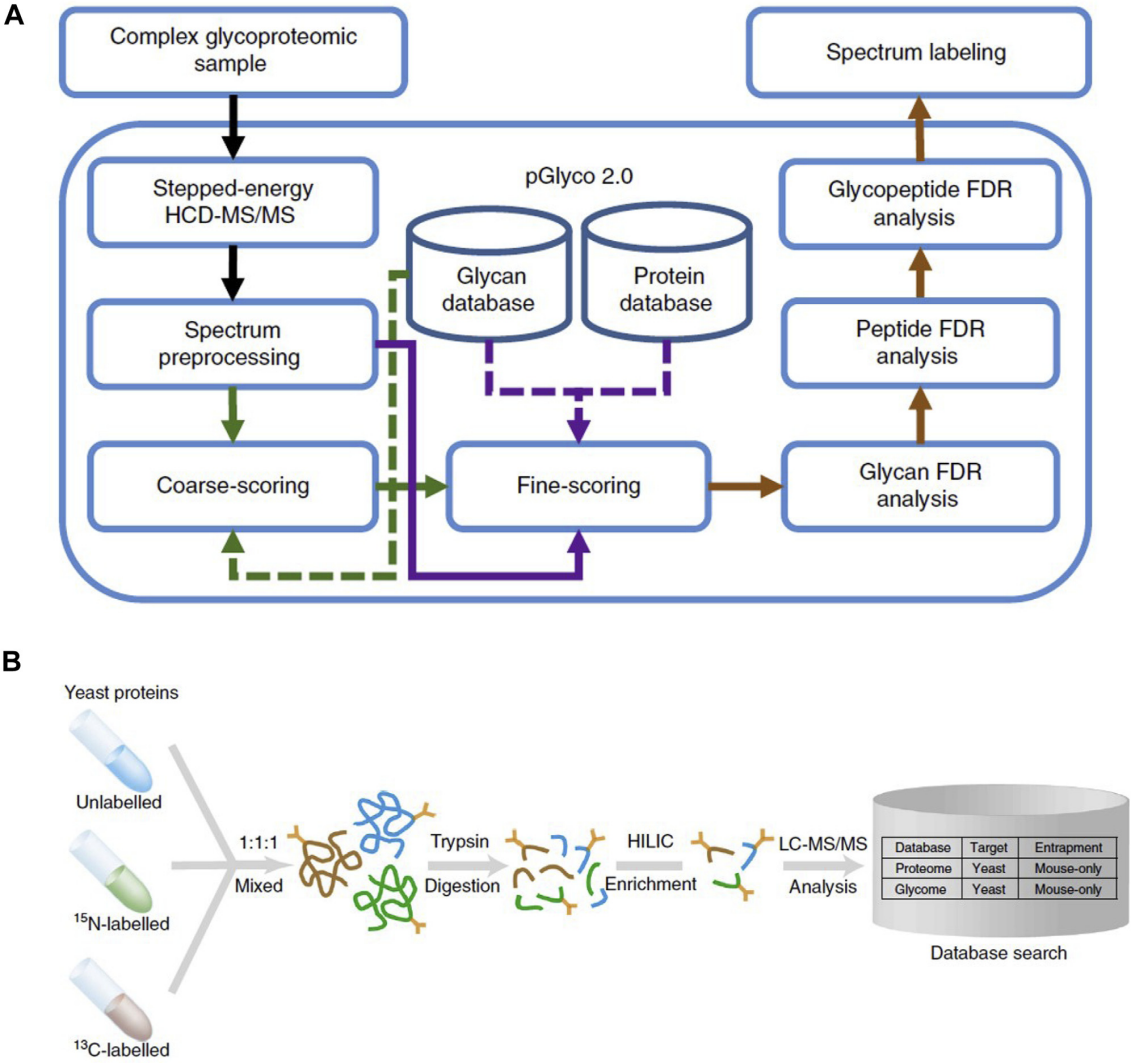
**A dedicated software pGlyco 2.0 for site-specific glycan interpretation.***A*, the flowchart of pGlyco 2.0. *B*, the FDR validation workflow. Reprinted with permission from Ref (26), under Creative Commons Attribution 4.0 International License (https://creativecommons.org/licenses/by/4.0/). FDR, false discovery rate.

Researchers also realized that an FDR evaluation at all three levels of matches to glycans, peptides, and glycopeptides is needed for comprehensive quality control (66). GP Finder (12) was a pioneer program attempting to resolve the problem of glycan decoy database construction. This software mainly focuses on identifying glycopeptides in simple samples. The issues with the mathematical model and algorithm of FDR for glycopeptide identification remained unsolved until pGlyco developed a novel target-decoy approach to estimate the FDR of the glycan identification. The scoring scheme for glycan identification by pGlyco was a revised version of the previously reported algorithm for the CID-MS/MS spectral analysis of glycopeptides (40). pGlyco 2.0 conducts comprehensive quality control, including an FDR analysis of glycans and peptides, and uses a new model for the glycopeptide FDR estimation (Fig. 2*A*). In addition, a new quantitative analysis pipeline using metabolically labeled glycoproteome samples was established to validate the FDR reported by the pGlyco 2.0 (Fig. 2*B*) (26).

## Mass Spectrum Annotation and Result Display

Automated annotation of glycopeptide spectra and a proper result display greatly facilitate subsequent analyses of intact glycopeptides. However, a range of previously developed software tools, including many well-designed search engines such as Sweet-Heart (54), ArMone (41), and GPQuest (42), do not include these functions. Some subsequently developed software tools, such as pGlyco (61), glyXtoolMS (67), and GPSeeker (68), conduct simple mass spectrum annotation steps. A web server, MAGIC-web (69), which was established by the MAGIC (44) software development team, enables mass spectrum annotation and glycan structure visualization. This function was designed as one of the functional modules on the web server and not integrated into the search engine of MAGIC and MAGIC+, requiring users have to complete additional operation themselves.

**Fig. 3:**
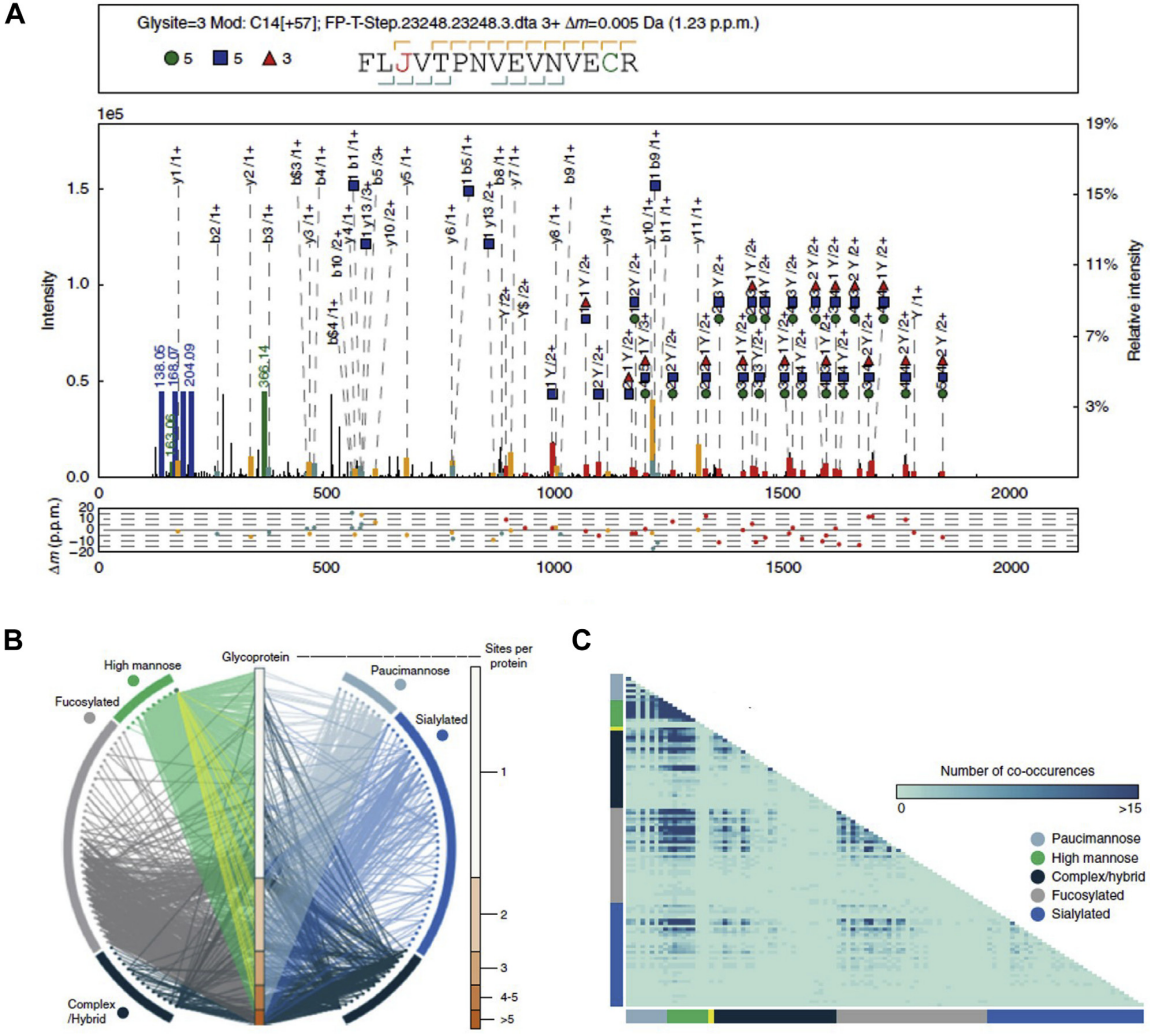
**Glycopeptide spectrum annotation and result display.***A*, automated annotation of glycopeptide spectra by pGlyco 2.0. *B*, glycoprotein–glycan network maps. *C*, a glycan co-occurrence heat map represents the number of times glycan pairs appeared together at the same glycosite. Reprinted with permission from Ref (26) and Ref (35), under Creative Commons Attribution 4.0 International License (https://creativecommons.org/licenses/by/4.0/).

The dedicated search engine pGlyco 2.0 (26) considered that issue. In pGlyco 2.0, all identified spectra were automatically annotated and displayed by the software tool gLabel embedded in pGlyco 2.0, which greatly facilitates manual verification and data analysis (Fig. 3*A*). Another impressive tool for visualizing results was proposed by Riley *et al.* (35). The authors developed several approaches to visualize identified glycopeptides and explore profiles of heterogeneity present at multiple levels of proteomic information, from glycosites to subcellular regions (Fig. 3, *B* and *C*), using a dataset identified by an activated ion electron transfer dissociation–enabled method, which proved useful for analyzing intact glycopeptides in future studies. In addition, some specialized glycopeptide mass spectrum annotation and visualization tools, such as PepSweetener (70) and gpAnnotate (71), were also designed to facilitate the identification and analysis of results.

## Software Applications

Advanced and dedicated software tools for high-throughput glycopeptide identification and efficient identification strategies have facilitated the increase in the N-glycoproteome coverage in recent years (48, 62, 72, 73, 74, 75, 76, 77, 78, 79, 80, 81). The intact glycopeptide identification scale increased by three orders of magnitude from 2010 to the present. Before 2014, only tens to hundreds intact glycopeptides were identified because almost all identification strategies and software tools were only suitable for glycopeptide analysis in simple or standard glycoprotein sample and the analytical throughput was low. For example, only 45 N-glycopeptides were identified using GlycoPep Grader in 2012 (13). Pompach *et al.* (15) identified 57 glycopeptides in haptoglobin and 14 glycopeptides using GlycoPeptide Search. The development of advanced search tools, more powerful instrumentation, and well-designed MS acquisition methods have increased the scale of identification from approximately 100 (42, 54, 82) to more than 1000 (40, 53, 76, 83). One of the most impressive and representative studies was published in 2016 by Sun *et al.* (83), who used GPQuest software and described an innovative method called solid-phase extraction of N-linked glycans and glycosite-containing peptides for N-glycopeptide identification. The authors successfully identified 1562 N-glycopeptides in different cell lines using the GPQuest software they developed in 2015 (42), which was a highlight and substantial achievement at that time.

In 2017, a major breakthrough was achieved by pGlyco 2.0 in the global characterization of intact N-glycopeptides (10, 26). The powerful search engine pGlyco 2.0 with comprehensive quality control and the ingenious experimental strategy identified more than 10,000 glycopeptides and reached the deepest and largest scale ever reported. A total of 10,009 intact N-glycopeptides were identified in five mouse tissues. The generic, precise, and open-access characters (download freely from http://pfind.ict.ac.cn/software/pGlyco/index.html) attracted increasing attention to pGlyco 2.0, and this method adopted by an increasing number of studies (28, 84, 85, 86, 87, 88, 89, 90, 91, 92, 93, 94, 95). For instance, pGlyco 2.0 was used to identify 3524 intact N-glycopeptides in the APP/PS1 mouse model of Alzheimer’s disease and WT mice. Based on the further integration and identification of N-glycopeptides, proteome and lectin microarray data for glycan epitopes and dysfunctions of N-glycoproteins were shown to affect the development of Alzheimer’s disease (27). Using pGlyco 2.0, Zhang *et al.* (92). established an in-depth N-glycopeptide profile of human plasma and identified 1644 intact N-glycopeptides.

Byonic has become commercially available around 2012 (48). Since then, it has been widely used in glycopeptide identification (96, 97, 98, 99, 100). In addition, Byonic enables the user to perform glycopeptide searches by specifying glycan masses using Byonic’s usual modification fine-control mechanism, which greatly facilitates many studies (101, 102).

## Conclusions and Future Perspectives

Powerful software tools are tremendously advancing the depth and precision of the identification of intact glycopeptides at a rapid rate and will greatly facilitate a comprehensive and accurate understanding of the role of glycosylation in organisms. Here, we reviewed the recent advances in software tools for MS-based identification of intact glycopeptides, typically for intact N-glycopeptides (Table 1). Throughout its development in the last decade, the field of N-glycopeptide analysis recently advanced in the last 5 years, changing the status of software development that lagged behind several years ago (103).

Glycopeptide analysis is moving toward the next stage of more comprehensive and quantitative identification. In our view, however, several problems must be solved in this process. The first is to develop dedicated strategies and search engines for intact O-glycopeptides. The problem of identifying O-glycopeptides are many folds, such as lack of efficient enrichment method, hard to obtain informative O-glycopeptide mass spectra. Although strategies and interpretation tools for O-glycoepeptides are emerging (104, 105, 106), they are immature and not as generic and precise as software tools for N-glycopeptides. The second issue to be addressed is the accurate site-specific localization of the glycan, particularly for O-glycopeptides, because several glycosylation sites occur adjacently in one digested O-glycopeptides, which presents a substantial challenge for both MS identification and software interpretation. The third is that generic software tools supporting both MS and tandem MS quantitation of intact glycopeptides urgently needed, which is extremely important for explorations of the roles of glycosylation in different biological and pathological stages. Fourth and importantly, because only the composition and a small degree of topology (such as fucose being on chitobiose core, etc.) can be generated by the current glycopeptide analysis approaches, the analyses of glycan structures and glycan isoforms on glycopeptides are additional and important challenges due to the inherent complexity of glycans. Extensive developments in techniques and software tools are needed to achieve more exquisite and superb tools for intact glycopeptide characterization and are anticipated in the next 5 to 10 years.

## Conflict of interest

The authors declare no competing interests.
